# *AtSOFL1* and *AtSOFL2* Act Redundantly as Positive Modulators of the Endogenous Content of Specific Cytokinins in *Arabidopsis*

**DOI:** 10.1371/journal.pone.0008236

**Published:** 2009-12-09

**Authors:** Jingyu Zhang, Radomira Vankova, Jiri Malbeck, Petre I. Dobrev, Yunyuan Xu, Kang Chong, Michael M. Neff

**Affiliations:** 1 Key Laboratory of Photosynthesis and Molecular Environmental Physiology, Institute of Botany, Chinese Academy of Sciences, Beijing, China; 2 Laboratory of Hormonal Regulations in Plants, Institute of Experimental Botany, Prague, Czech Republic; 3 Department of Crop and Soil Sciences, Washington State University, Pullman, Washington, USA; Ecole Normale Superieure, France

## Abstract

**Background:**

Although cytokinins have been known for decades to play important roles in the regulation of plant growth and development, our knowledge of the regulatory mechanism of endogenous content of specific cytokinins remains limited.

**Methodology/Principal Findings:**

Here, we characterized two SOB five-like (SOFL) genes, *AtSOFL1* and *AtSOFL2*, in *Arabidopsis* (*Arabidopsis thaliana*) and showed that they acted redundantly in regulating specific cytokinin levels. Analysis of the translational fusion *AtSOFL1:AtSOFL1-GUS* and *AtSOFL2:AtSOFL2-GUS* indicated that *AtSOFL1* and *AtSOFL2* exhibited similar expression patterns. Both proteins were predominantly expressed in the vascular tissues of developing leaves, flowers and siliques, but barely detectable in roots and stems. Overexpression of either *AtSOFL1* or *AtSOFL2* led to increased cytokinin content and obvious corresponding mutant phenotypes for both transgenic seedlings and adult plants. In addition, overexpression and site-directed mutagenesis experiments demonstrated that the SOFL domains are necessary for *AtSOFL2*'s overexpression phenotypes. Silencing or disrupting either *AtSOFL1* or *AtSOFL2* caused no obvious developmental defects. Endogenous cytokinin analysis, however, revealed that compared to the wild type control, the *SOFL1-RNAi62 sofl2-1* double mutant accumulated lower levels of *trans*-zeatin riboside monophosphate (tZRMP) and *N^6^*-(Δ^2^-isopentenyl)adenosine monophosphate (iPRMP), which are biosynthetic intermediates of bioactive cytokinins. The double mutant also displayed decreased response to exogenous cytokinin in both callus-formation and inhibition-of-hypocotyl-elongation assays.

**Conclusions/Significance:**

Taken together, our data suggest that in plants AtSOFL1 and AtSOFL2 work redundantly as positive modulators in the fine-tuning of specific cytokinin levels as well as responsiveness.

## Introduction

Cytokinins are classic phytohormones that play important roles in the regulation of plant growth and development [1], [2], [3], [4]. Recent work has revealed the major steps of cytokinin synthesis in *Arabidopsis* (*Arabidopsis thaliana*) [2], [3], [5], [6]. The first step is catalyzed by adenosine phosphate-isopentenyltransferase (IPT). IPTs are encoded by a small gene family in plants, with nine and eight members in *Arabidopsis* and rice (*Oryza sativa*), respectively [7], [8], [9]. Another cytokinin biosynthetic gene in petunia (*Petunia hybrida*), *Shooting* (*Sho*), was isolated in an activation-tagging mutant screen [10].

In higher plants, the major initial products from the first step of cytokinin biosynthesis are *N^6^*-(Δ^2^-isopentenyl)adenine (iP) nucleotides, which are then converted into *trans*-zeatin (tZ) nucleotides by the cytochrome P450 mono-oxygenases CYP735A1 and CYP735A2 [11]. Based on deuterium labeling and mass spectrometry experiments, Åstot *et al.* proposed the possibility of an alternative, *N^6^*-(Δ^2^-isopentenyl)adenosine monophosphate (iPRMP)-independent biosynthetic pathway for zeatin (Z)-type cytokinins, in which tZ could be formed by the direct addition of a hydroxylated side chain to the adenine moiety [12]. To become biologically active, iP- and tZ-nucleotides need to be converted to free-base forms by dephosphorylation and deribosylation. Recently, a novel pathway that directly releases active cytokinins from their nucleotides was identified [13]. Thus, it is likely that there are at least two cytokinin activation pathways in plants.

Cytokinin oxidase/dehydrogenase (CKX) catalyzes the irreversible degradation of cytokinins and in many plant species is responsible for the majority of metabolic cytokinin inactivation [2], [9], [14], [15]. The *CKX* gene was first cloned from maize by Houba-Hérin *et al*. [16] and Morris *et al*. [17], then from *Arabidopsis* [15], [18] and orchids [19]. The *Arabidopsis AtCKX* gene family has seven members (*AtCKX1* to *AtCKX7*) [14]. Stability of cytokinins, determined by their affinity to metabolic enzymes, also has an effect on their biological activity. The physiologically active cytokinins, tZ and iP, are readily degraded by CKXs from various plant species [18], [20], whereas *cis*-zeatin (cZ) is generally less amenable to catalysis [18]. Dihydrozeatin (DZ) and aromatic cytokinins are resistant to CKXs because CKXs-mediated degradation requires the double bond in the substrate's isoprenoid side chain [21]. Recent studies have shown that Uridine-Ribohydrolase 1 can cleave the cytokinin derivative isopentenyladenine-riboside, and is therefore probably involved in cytokinin degradation [22].

In *Arabidopsis*, both the synthesis and the degradation of cytokinins are regulated by members of small gene families. These family members have distinct spatial expression patterns and responsiveness to hormones, suggesting that specific developmental and physiological functions are fulfilled by different genes and that the endogenous cytokinin content is under strict control *in planta* [23], [24], [25]. Although the identification of cytokinin metabolic enzymes sheds light on the molecular basis of cytokinin metabolism, the overall regulatory network controlling cytokinin biosynthesis and catabolism is still unclear, with some metabolic steps remaining uncovered [2], [3], [6], [26].

The SOB five-like (SOFL) family is a group of novel plant-specific proteins with high sequence similarity and similar protein structures. Although overexpression analysis of one of its members, Activation-Tagged Suppressor of *phyB*-4 #5 (SOB5), suggests an involvement in cytokinin-mediated plant development [27], no loss-of-function mutant phenotype was observed, most likely due to the functional redundancy among the SOFL proteins. To further study the function of SOFLs and their potential role in the cytokinin metabolism, we characterized both overexpression transgenic plants and loss-of-function mutants for the two *Arabidopsis SOFLs*, *AtSOFL1* and *AtSOFL2*, which show higher similarity to each other than to *SOB5* and *SOFLs* from other plant species. Our data demonstrate that these two SOFL proteins act redundantly to modulate specific cytokinin levels as well as responsiveness.

## Results

### Overexpression of *AtSOFL1* and *AtSOFL2* Confers Semi-Dwarf Phenotypes

**Figure 1:**
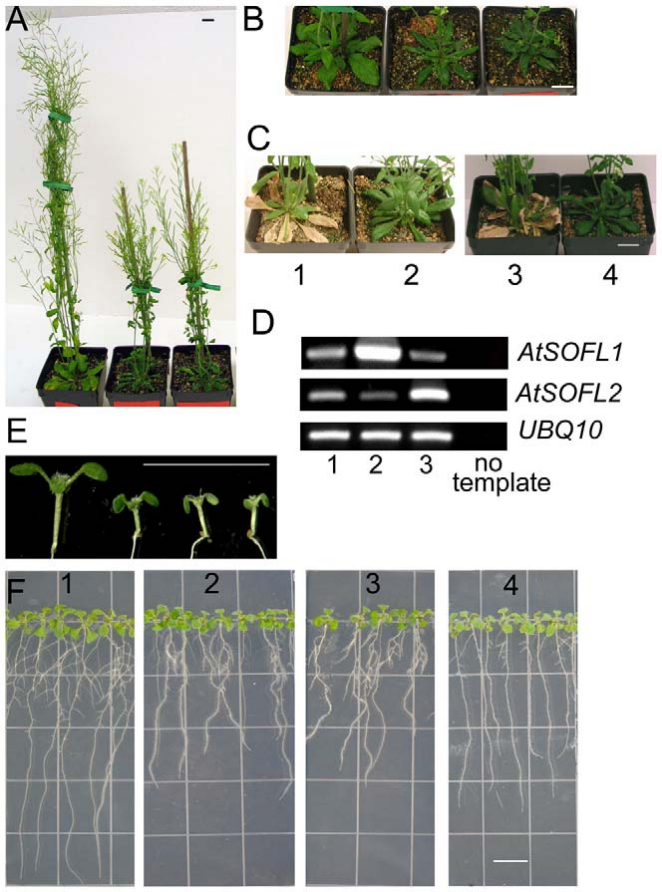
Phenotypes of transgenic plants overexpressing *AtSOFL1* or *AtSOFL2*. A, Seven-week-old Col-0, transgenic plants expressing *35S:AtSOFL1* and *35S:AtSOFL2* are shown from left to right. B, Five-week-old Col-0, transgenic plants expressing *35S:AtSOFL1* or *35S:AtSOFL2* are shown from left to right. C, Col-0 (C1, C3) and transgenic plants expressing *35S:AtSOFL1* (C2) and *35S:AtSOFL2* (C4) were grown for nine weeks. All the plants were grown under standard greenhouse conditions. Scale bar  = 10 mm. D, RT-PCR analysis of *35S:AtSOFL*-expressing plants. Total RNA was isolated from rosette leaves of seven-week-old Col-0 (D1) and transgenic plants expressing *35S:AtSOFL1* (D2) and *35S:AtSOFL2* (D3). PCR was performed on cDNA using primers specific for the *AtSOFL1* or *AtSOFL2* gene for 28 or 26 cycles, respectively. The *ubiquitin10* (*UBQ10*) cDNA, amplified for 22 cycles, was used as control to normalize the amount of cDNA in each of the samples. E, Six-day-old Col-0 and transgenic seedlings expressing the *35S:AtSOFL1* or *35S:AtSOFL2* construct as well as Col-0 seedlings grown on medium with 0.1 µM benzyladenine (BA) are shown from left to right. F, Six-day-old vertically-grown Col-0 (F1) and transgenic seedlings expressing the *35S:AtSOFL1* (F2) or *35S:AtSOFL2* (F3) construct as well as Col-0 seedlings grown vertically on medium with 0.01 µM BA (F4) are shown from left to right. All the seedlings were grown on half-strength MS medium with or without BA. Scale bar  = 10 mm.

The SOFL proteins exist in both dicots and monocots [27]. Overexpression of *SOB5* in *Arabidopsis* confers a dwarf phenotype [27].To investigate whether the two *Arabidopsis SOFL* genes, *AtSOFL1* (At1g26210) and *AtSOFL2* (At1g68870), have similar functions, each was overexpressed under the control of the cauliflower mosaic virus (CaMV) 35S promoter. Transgenic plants expressing *35S:AtSOFL1* and *35S:AtSOFL2* showed similar adult phenotypes (Figure 1A–C). The overexpression of either gene generated semi-dwarf plants with reduced apical dominance. Compared with wild-type plants, they had smaller leaves as well as underdeveloped root system and their life cycles were approximately 2 weeks longer (Figure 1A–C, F). These are characteristic phenotypes observed for transgenic plants expressing the *Agrobacterium tumefaciens ipt* gene, which encodes an enzyme that catalyzes the first step in the cytokinin biosynthetic pathway [28], [29], [30]. Although *sob5-D*, an activation-tagging mutant overexpressing the *SOB5* gene, also shows similar phenotypes [27], there were clear differences between *sob5-D* and *35S:AtSOFL1*, *35S:AtSOFL2* transgenic plants. When the *AtSOFL1*, *AtSOFL2* and *SOB5* genes were overexpressed at similar levels, *35S:AtSOFL1*, *35S:AtSOFL2* transgenic plants still maintained some degree of apical dominance and were obviously much taller than *sob5-D*, which almost totally lost its apical dominance (Figure 1A, D) [27]. *sob5-D* plants have curled-down adult leaves [27], which were rarely observed for *35S:AtSOFL1* and *35S:AtSOFL2* transgenic plants (Figure 1A).

The data presented above suggest that the phenotypic changes of *35S:AtSOFL1* and *35S:AtSOFL2* expressing plants are caused by the increase of endogenous cytokinin levels. To test this hypothesis, we grew *35S:AtSOFL1*, *35S:AtSOFL2* and wild-type seedlings on medium with or without cytokinin. The results indicated that the transgenic seedling phenotypes caused by overexpressing *AtSOFL1* or *AtSOFL2* could largely be phenocopied by supplying wild-type seedlings with exogenous cytokinin (Figure 1E, F). Both transgenic seedlings and cytokinin-treated wild-type controls showed epinastic cotyledons (Figure 1E) and inhibited root development, including shorter main roots and fewer lateral roots (Figure 1F, Table S1).

### The *35S:AtSOFL1* and *35S:AtSOFL2* Transgenic Plants Have Elevated Endogenous Cytokinin Levels

To investigate whether the endogenous cytokinin levels of *35S:AtSOFL1* and *35S:AtSOFL2* transgenic plants were increased, cytokinin content was measured in the aerial parts and roots of transgenic seedlings overexpressing *AtSOFL1* or *AtSOFL2*. Results from three independent experiments showed that both *35S:AtSOFL1* and *35S:AtSOFL2* transgenic seedlings accumulated higher amounts of *trans*-zeatin riboside (tZR) in the whole seedling and iPRMP in the aerial tissue. Levels of the cytokinin N-glucosides *trans*-zeatin 7-glucoside (tZ7G) and *cis*-zeatin 7-glucoside (cZ7G) were increased mainly in the roots of both *35S:AtSOFL1* and *35S:AtSOFL2* seedlings, whereas the level of *N^6^*-(Δ^2^-isopentenyl)adenine 7-glucoside (iP7G) was increased in both aerial and root tissue of transgenic seedlings (Table 1). In these samples, the levels of tZ, cZ, iP, *cis*-zeatin riboside (cZR) and *N^6^*-(Δ^2^-isopentenyl)adenosine (iPR) were below the detection limit. These data suggest that AtSOFL1 and AtSOFL2 are involved in regulating specific cytokinin levels in *Arabidopsis*. The *35S:AtSOFL1* and *35S:AtSOFL2* seedlings showed similar, but different altered cytokinin patterns (Table 1), suggesting that the functions of AtSOFL1 and AtSOFL2 are not exactly the same.

**Table 1:** Cytokinin content in the aerial portions and roots of wild-type (WT) and transgenic seedlings overexpressing *AtSOFL1* (OX-SOFL1) or *AtSOFL2* (OX-SOFL2).

	Cytokinin content (pmol g^−1^ FW)			
Cytokinin Metabolites	WT	Control^§^	OX-SOFL1	OX-SOFL2
**Aerial Tissue**				
tZR	6.80±2.61	4.27±1.18	17.43±5.74*	14.00±1.16 *
tZRMP	18.33±0.67	10.10±2.55	16.37±5.33	17.00±4.36
cZRMP	2.87±0.64	2.53±0.49	3.47±0.37*	2.70±0.70
iPRMP	4.50±0.25	3.80±0.73	6.33±1.18*	7.13±1.22*
tZ7G	150.00±5.77	135.00±12.81	164.67±45.70	156.67±17.64
cZ7G	44.00±5.51	38.67±2.33	46.00±8.00	53.67±9.53*
iP7G	73.67±4.33	70.00±5.13	104.00±11.37*	102.33±9.58*
**Roots**				
tZR	8.40±2.98	8.53±2.24	14.67±2.03*	20.67±5.36*
tZRMP	8.23±3.99	11.76±3.11	8.10±1.95	10.80±3.25
cZRMP	0.60±0.38	0.80±0.21	0.67±0.33	1.17±0.62*
iPRMP	1.40±0.64	1.77±0.35	1.37±0.32	3.63±1.59*
tZ7G	62.67±12.20	51.33±4.67	82.33±8.87*	93.33±27.44*
cZ7G	44.00±6.08	48.33±11.86	77.67±4.91**	105.67±34.82*
iP7G	12.27±6.70	14.00±1.53	30.33±2.91*	39.67±12.14*

Cytokinin quantification was performed on aerial tissue and roots of two-week-old seedlings. The results are based on three independent experiments and resulting data are expressed as means ± standard error. (*) equals P<0.5 and (**) equals P<0.05 from a Student's unpaired two-tailed *t* test comparing the mutant and the wild-type seedlings. The levels of *trans*-zeatin (tZ), *cis*-zeatin (cZ), *N^6^*-(Δ^2^-isopentenyl)adenine (iP), *cis*-zeatin riboside (cZR) and *N^6^*-(Δ^2^-isopentenyl)adenosine (iPR) were below the detection limit. §, transgenic plants expressing the empty pCHF3 vector, used as control for cytokinin measurement; tZR, *trans*-zeatin riboside; tZRMP, *trans*-zeatin riboside monophosphate; tZ7G, *trans*-zeatin 7-glucoside; cZRMP, *cis*-zeatin riboside monophosphate; cZ7G, *cis*-zeatin 7-glucoside; iPRMP, *N^6^*-(Δ^2^-isopentenyl)adenosine monophosphate; iP7G, *N^6^*-(Δ^2^-isopentenyl)adenine 7-glucoside.

### The SOFL Domains Are Necessary for *AtSOFL2*'s Overexpression Phenotypes

**Figure 2:**
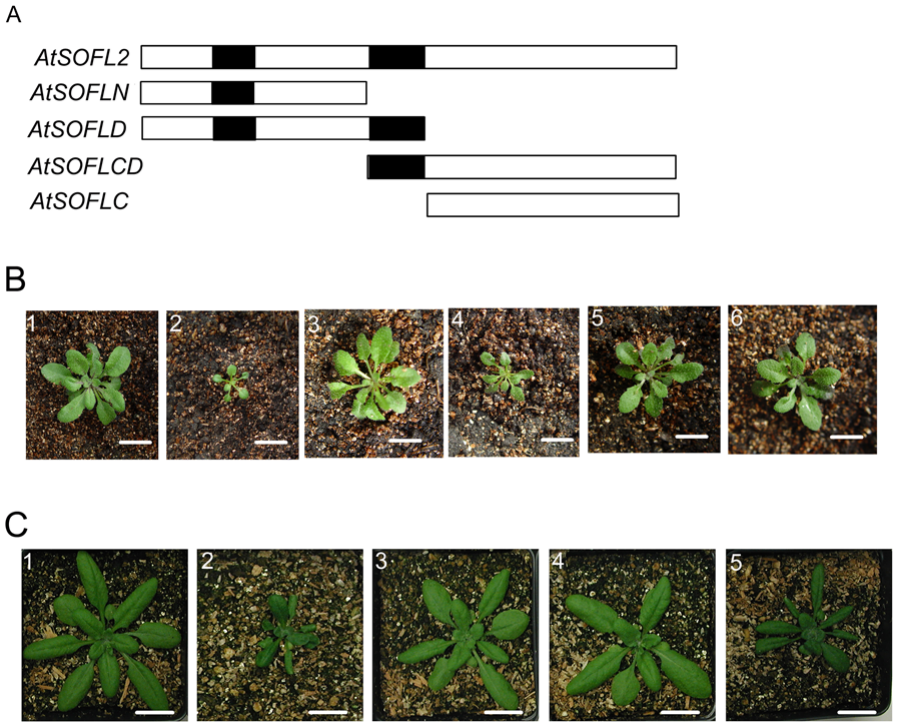
Phenotypes of transgenic plants overexpressing different domains of *AtSOFL2* and the full-length *AtSOFL2* gene with point mutations. A. The schematic diagram of *AtSOFL2* and the constructs used for overexpression experiment, including *AtSOFLN, AtSOFLD, AtSOFLCD* and *AtSOFLC*. Empty and solid rectangles represent protein-coding regions and the SOFL domains of AtSOFL2, respectively. B. The wild-type (B1), *35S:AtSOFL2* plants (B2) and transgenic plants overexpressing *AtSOFLN* (B3), *AtSOFLD* (B4), *AtSOFLCD* (B5) and *AtSOFLC* (B6) were grown for one month and a half in the green house. Scale bar  = 1.5 cm. C. The wildtype (C1), *35S:AtSOFL2* plants (C2) and transgenic plants overexpressing *AtSOFL2* gene with point mutations T21I (C3), D80N (C4) and D52N (C5) were grown for one month in the greenhouse. Scale bar  = 1.5 cm.

There are two conserved domains, SOFLI and SOFLII, among SOFL proteins. The regions outside the SOFL domains are diverged, especially at C-terminal ends of the proteins. To investigate the function of different SOFL domains, we generated a series of constructs including *AtSOFLN, AtSOFLD, AtSOFLCD* and *AtSOFLC*, which expressed different N-terminal or C-terminal regions of AtSOFL2, respectively (Figure 2A, B). All the constructs were expressed under the control of the CaMV 35S promoter and separately transformed into wild-type *Arabidopsis* plants. The resulting transgenic plants expressing *AtSOFLN, AtSOFLC* and *AtSOFLCD* showed the phenotypes of wild-type plants (Figure 2, B3, B5-B6), suggesting that both SOFLI and SOFLII domains are needed for the overexpression phenotype of *AtSOFL2*. Transgenic plants expressing *AtSOFLD* showed similar (Figure 2, B4), but less severe mutant phenotypes compared to the *35S:AtSOFL2* transgenic plants, implying that the C-terminal end of AtSOFL2 is also required for its function.

To further examine the requirement of some highly conserved residues for AtSOFL2's function, site-directed mutagenesis experiment was performed. A series of site-directed point mutations were generated for the *AtSOFL2* gene. Transgenic plants overexpressing the *AtSOFL2* gene with mutations in the conserved SOFL domains, T21I and D80N, exhibited wild-type phenotype (Figure 2, C3–C4), suggesting that these conserved amino acid residues are important for AtSOFL2's function. When the less conserved amino acid residue, D52N, was mutated, the transgenic plants overexpressing the mutated gene exhibited mutant phenotypes similar to those observed for *35S:AtSOFL2* transgenic plants (Figure 2, C5), suggesting that this amino acid residue is less important for *AtSOFL2*'s overexpression phenotype.

### Expression Patterns of *AtSOFL1* and *AtSOFL2*

**Figure 3:**
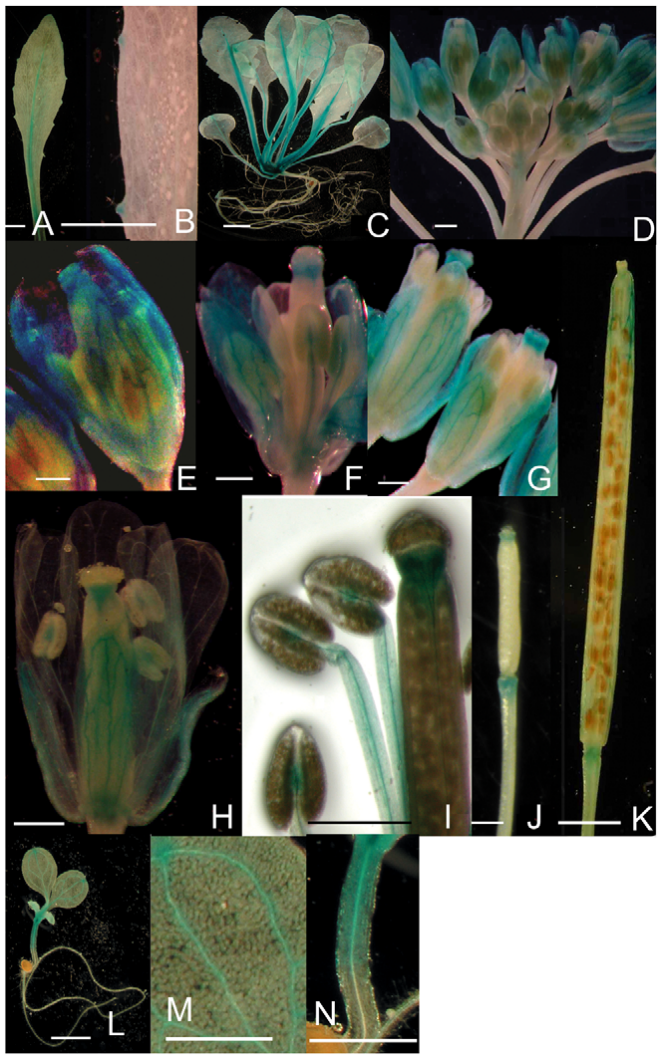
Histochemical localization of AtSOFL1 and AtSOFL2 in *Arabidopsis*. Transgenic plants expressing the *AtSOFL1:AtSOFL1-GUS* (A–F, H–N) or *AtSOFL2:AtSOFL2-GUS* (G) constructs were grown on half-strength Murashige and Skoog (MS) medium for one (L–N) or two weeks (C) or in soil for four (A–B), six (D–J) or eight weeks (K) in the greenhouse. A, Rosette leaf showing GUS staining in the midrib and hydathodes. B, Close-up of hydathodes. C, Whole plant. D, Inflorescence. E, Unopened flower showing staining in the sepal vasculature. F, Unopened flower that was opened to show staining in the vascular tissue of stamens and the pistil tip. G, Flowers for *AtSOFL2:AtSOFL2-GUS* transgenic plants. H, Fully-opened flower. I, Close-up of the pistil tip and stamens of a fully-opened flower. J, Young silique. K, Mature silique. L, One-week-old seedlings. M, Close-up of cotyledon. N, Close-up of hypocotyl. Scale bar  = 500 µm.

The expression patterns of *AtSOFL1* and *AtSOFL2* in wild-type plants were analyzed via translational fusions with the β-glucuronidase (GUS) reporter gene. *Arabidopsis* plants (Col-0) were transformed with constructs encoding AtSOFL1-GUS or AtSOFL2-GUS translational fusion proteins that were expressed using the corresponding promoter region of the *AtSOFL1* or *AtSOFL2* gene. Histochemical analysis of both *AtSOFL1:AtSOFL1-GUS* and *AtSOFL2:AtSOFL2-GUS* transgenic plants showed that GUS activity was mainly present in the vascular tissues of leaves, flowers and siliques, but barely detectable in roots and stems (Figure 3). These results are consistent with the expression data for *AtSOFL1* in the massively parallel signature sequencing (MPSS) database (http://mpss.udel.edu/at/) that was constructed by Nakano *et al.* [31]. No MPSS expression data is available for *AtSOFL2*.

For *AtSOFL1:AtSOFL1-GUS*-expressing transgenic plants, GUS activity was strong in the midrib of leaf veins of developing leaves, but was relatively weak in the hydathode regions (Figure 3A–C). GUS activity was undetectable in young flower buds, but was present in pistil tips and the vascular tissue of stamens and sepals of developing flower buds (Figure 3D–F). In fully opened flowers, GUS activity was detected in the vascular bundles between the two anther locules, the central vascular cylinders of the filaments, tips and bases of the pistils, and sepal vascular tissue (Figure 3H, I). In pistil tips, GUS activity was stronger in vascular tissues (Figure 3I). GUS activity was also observed at the tips and bases of developing siliques (Figure 3J). When the siliques matured, GUS activity was still present at these positions, but it was restricted to the vascular tissues at the silique tips (Figure 3K).

The expression pattern of *AtSOFL1:AtSOFL1-GUS* and *AtSOFL2:AtSOFL2-GUS* was also examined in 6-day-old seedlings. For both *AtSOFL1:AtSOFL1-GUS* and *AtSOFL2:AtSOFL2-GUS*, GUS activity was strong in the hydathode region of cotyledons as well as the upper part of hypocotyls and was weak in the vascular tissue of cotyledons. No GUS activity was detected in the roots (Figure 3L–N).

The *AtSOFL2:AtSOFL2-GUS* translational fusion showed an expression profile similar to that of the *AtSOFL1:AtSOFL1-GUS* during plant development except that no obvious GUS activity was detected in the anthers and pistil bases of opened *AtSOFL2:AtSOFL2-GUS* flowers (Figure 3G). Both *AtSOFL1:AtSOFL1-GUS* and *AtSOFL2:AtSOFL2-GUS* transgenic plants mainly showed GUS activity in the vascular tissue (Figure 3), suggesting that the function of AtSOFL1 and AtSOFL2 may have some connection with cytokinin transport.

### Isolation of an Insertion Mutant for *AtSOFL2*

To study phenotypes conferred by loss-of-function mutations in the *SOFL* genes, we screened the existing collection of insertion mutants of *AtSOFL1* or *AtSOFL2*. We could not identify any insertions in *AtSOFL1*, but obtained a mutant (*sofl2-1*) carrying a T-DNA insertion in the *AtSOFL2* open reading frame from the *Arabidopsis* Biological Resource Center (ABRC). This mutant is likely to be a null allele because no full-length *AtSOFL2* transcript was detected by RT-PCR analysis in RNA samples from *sofl2-1* (Figure S1). No obvious mutant phenotype, however, was observed for the *sofl2-1* mutant (data not shown), suggesting that there may be functional redundancy between *AtSOFL1* and *AtSOFL2*.

### Silencing of *AtSOFL1* Gene in the *sofl2-1* Mutant Background

To further investigate the function of *AtSOFL1* and *AtSOFL2* in *Arabidopsis*, we generated a RNA interference (RNAi) construct to silence the *AtSOFL1* gene in the *sofl2-1* mutant background. A 471-bp region of the *AtSOFL1* gene containing 3′ coding and untranslated region was amplified by PCR and cloned into a binary vector pKANNIBAL in a hairpin manner, resulting in the RNAi construct pRNAi-SOFL1. This construct was first transformed into wild-type *Arabidopsis* plants. RT-PCR analysis showed that the expression level of *AtSOFL1* was dramatically reduced in the T1 transgenic plants, but no reduction was detected for the expression of the *AtSOFL2* gene (Figure S2). No obvious mutant phenotype was observed for the resulting *SOFL1-RNAi* lines (data not shown) and no obvious change was detected for its endogenous cytokinin content (Table S2).

**Figure 4:**
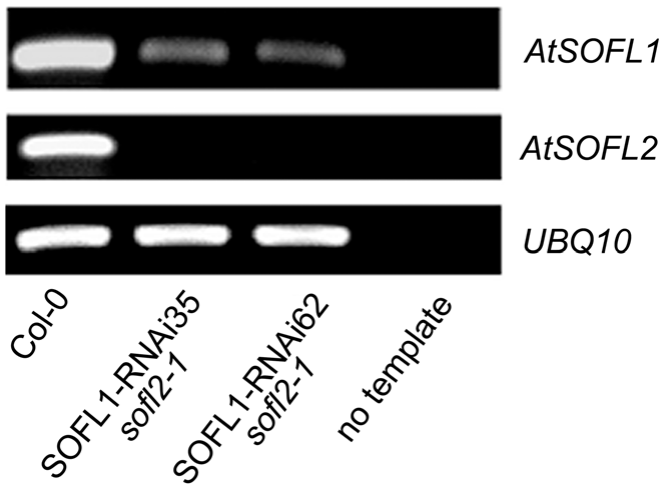
RT-PCR analysis of *SOFL1-RNAi sofl2-1* lines. Total RNA was isolated from seedlings grown under continuous white light for 5 days. PCR was performed on cDNA by amplifying with *AtSOFL1*-specific primers for 32 cycles and *AtSOFL2*-specific primers for 30 cycles. The *UBQ10* cDNA, amplified for 22 cycles, was used as control to normalize the amount of cDNA in each of the samples.

The pRNAi-SOFL1 construct was then transformed into the *sofl2-1* mutant and multiple homozygous lines with reduced expression of the *AtSOFL1* gene were isolated (Figure 4). Since multiple *SOFL1-RNAi sofl2-1* lines showed similar mutant phenotypes (Table S3), we describe below the *SOFL1-RNAi62 sofl2-1* as a representative line.

### The *SOFL1-RNAi62 sofl2-1* Line Has Reduced Endogenous Cytokinin Levels

It has been shown that the overexpression of *AtSOFL1* or *AtSOFL2* results in higher endogenous cytokinin content (Table 1). To further explore the role of *AtSOFL1* and *AtSOFL2* in cytokinin metabolism, the content of isoprenoid cytokinins were measured in 7-week-old *SOFL1-RNAi62 sofl2-1* and wild-type plants. Although the *SOFL1-RNAi* lines showed no obvious change for their endogenous cytokinin content (Table S2), clear changes were detected for the *SOFL1-RNAi62 sofl2-1* mutant. Based on three independent experiments, the most significant changes in the *SOFL1-RNAi62 sofl2-1* mutant was the decreased content of *trans*-zeatin riboside monophosphate (tZRMP) and iPRMP, which was about 5% and 3% of the wild type content, respectively (Table 2). There was also a slight increase (less than 2.5-fold) in the levels of cZ, *cis*-zeatin 9-glucoside (cZ9G), iP7G and *N^6^*-(Δ^2^-isopentenyl)adenine 9-glucoside (iP9G) (Table 2). No statistically significant differences were detected for the content of other cytokinin metabolites. Other *SOFL1-RNAi sofl2-1* lines exhibited similar changes for their endogenous cytokinin content (Table S3).

**Table 2:** Cytokinin content in the *SOFL1-RNAi62 sofl2-1* mutant and wild-type (WT) plants.

	Cytokinin content (pmol g^−1^ FW)	
Cytokinin Metabolites	WT	*SOFL1-RNAi62 sofl2-1*
tZ	0.03±0.01	0.02±0.01
tZR	2.05±0.33	3.08±0.97
tZRMP	13.10±4.44	0.20±0.20**
cZ	0.80±0.41	1.38±0.09*
cZR	14.77±14.12	23.83±4.80
cZRMP	2.68±0.90	2.85±0.25
iP	ND	ND
iPR	2.97±2.79	4.13±0.79
iPRMP	1.55±0.35	0.08±0.04**
tZ7G	31.83±3.77	29.33±0.67
tZ9G	10.00±1.75	9.93±0.59
tZROG	1.10±0.15	1.12±0.06
cZ9G	3.07±0.14	3.88±0.34*
DHZ7G	2.02±0.51	2.03±0.33
iP7G	10.12±1.83	17.00±0.50**
iP9G	0.27±0.02	0.62±0.04***

Cytokinin quantification was performed on rosette leaves from plants that were grown under short-day growth conditions for 3 weeks and moved to long-day growth conditions for 4 weeks. The results are based on three independent experiments and resulting data are expressed as means ± standard error. (*) equals P<0.5, (**) equals P<0.05 and (***) equals P<0.005 from a Student's unpaired two-tailed *t* test comparing the mutant and the wild-type plants. ND, not detectable. cZ, *cis*-zeatin; cZR, *cis*-zeatin riboside; tZ9G, *trans*-zeatin 9-glucoside; tZROG, *trans*-zeatin riboside *O*-glucoside; cZ9G, *cis*-zeatin 9-glucoside; DHZ7G, dihydrozeatin 7-glucoside; iP9G, *N^6^*-(Δ^2^-isopentenyl)adenine 9-glucoside. Other abbreviations are either defined in the text or in the legend of Table 1.

### The Cytokinin Sensitivity of the *SOFL1-RNAi62 sofl2-1* Line

**Figure 5:**
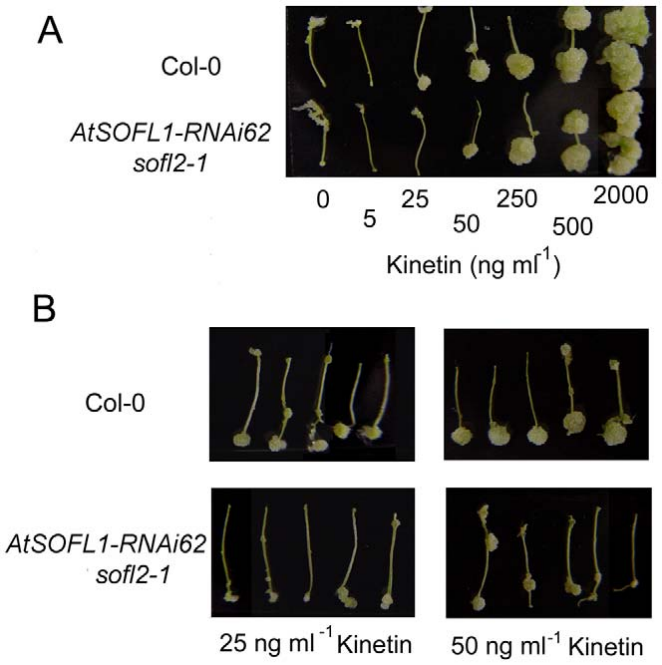
Response of *SOFL1-RNAi62 sofl2-1* and wild-type plants to exogenous cytokinins. Response of root explants from *SOFL1-RNAi62 sofl2-1* and wild-type seedlings to different cytokinin concentrations. The upper-part hypocotyl segments were cultured for 24 days on medium containing 10 ng ml^−1^ 2,4-dichlorophenoxyacetic acid (2,4-D) and different concentrations of kinetin (A) or at the concentration of 25 ng ml^−1^ and 50 ng ml^−1^ kinetin (B).

To investigate the potential role of AtSOFL1 and AtSOFL2 in cytokinin-mediated development, the sensitivity of the *SOFL1-RNAi62 sofl2-1* to exogenous cytokinins was examined with both callus-formation [32] and inhibition-of-hypocotyl-elongation assays [33]. Analysis of *AtSOFL1:AtSOFL1-GUS* and *AtSOFL2:AtSOFL2-GUS* indicated that *AtSOFL1* and *AtSOFL2* were expressed in the upper part of hypocotyls, but not in roots (Figure 3). Thus, a callus formation assay was performed with segments from the upper part of hypocotyls for the double mutant and the wild type control. The *SOFL1-RNAi62 sofl2-1* explants produced smaller callus than control explants at 25 and 50 ng ml^−1^ kinetin in this assay (Figure 5), suggesting that the double mutant exhibited less sensitivity to exogenous cytokinins.

**Figure 6:**
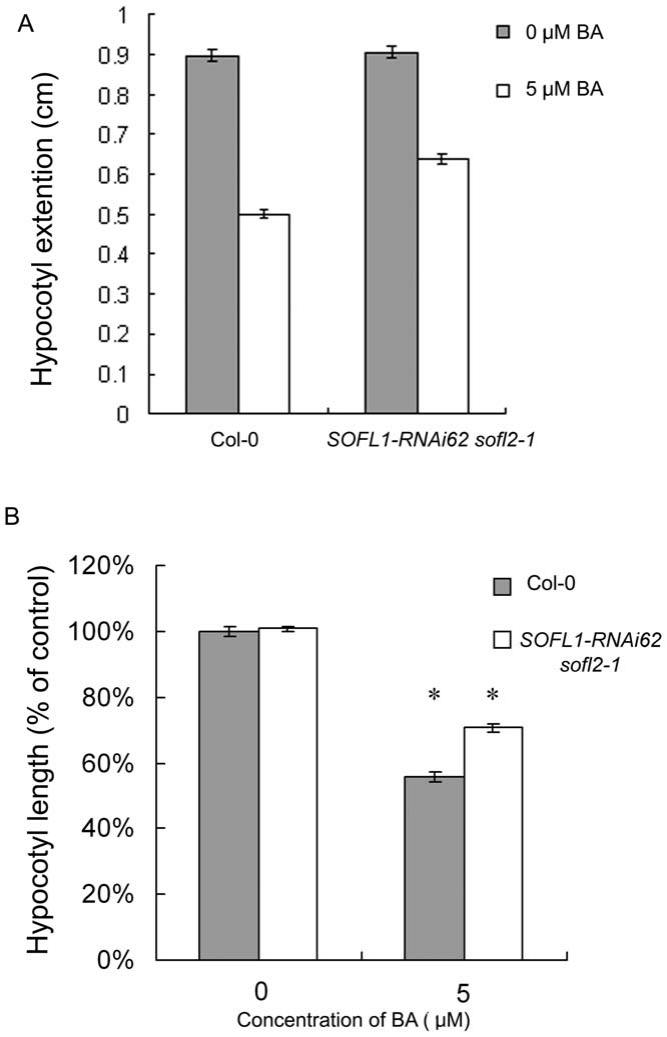
Effects of exogenous cytokinins on hypocotyl growth of dark-grown *SOFL1-RNAi62 sofl2-1* seedlings. Seedlings were grown for 6 days in the dark on MS medium. A, Hypocotyl length of 6-day-old seedlings, expressed as the mean ± S.E, n>25. B, Hypocotyl elongation expressed relative to the mean hypocotyl length of corresponding plants grown in the absence of BA. * equals P<0.05 from a Student's unpaired two-tailed *t* test comparing the mutant and the wild-type seedlings.

Hypocotyl elongation of wild-type plants in the dark is inhibited in the presence of cytokinin [34]. To further investigate the function of AtSOFL1 and AtSOFL2 in this cytokinin response, we measured hypocotyl elongation of the double mutant and control seedlings grown in the dark in the presence and absence of cytokinin (Figure 6). As was seen for wild-type seedlings, hypocotyls of the *SOFL1-RNAi62 sofl2-1* seedlings were shorter in the presence of cytokinin, but a reduced response was observed when compared to the wild type (P<0.05, Figure 6). This result is consistent with the decreased sensitivity shown by the double mutant in the callus formation assay.

## Discussion

AtSOFL1 and AtSOFL2 belong to the plant-specific SOFL protein family and have 59% amino acid identity to each other. The SOFL domains are necessary for them to perform their functions in plants (Figure 2). They show much lower amino acid identity (23%) to SOB5, another SOFL protein in *Arabidopsis* [27]. Analysis of translational fusions with the GUS reporter gene indicated that AtSOFL1 and AtSOFL2 have nearly identical expression patterns (Figure 3), which are different from SOB5's [27]. Overexpression of both *AtSOFL1* and *AtSOFL2* conferred semi-dwarf plants with increased cytokinin content (Figure 1, Table 1), suggesting that there is functional redundancy between these two genes. There are clear differences between *sob5-D* and *35S:AtSOFL1*, *35S:AtSOFL2* transgenic plants (Figure 1) [27], implying that functional divergence exists between *SOB5* and *AtSOFL1*, *AtSOFL2* genes. To determine the function of AtSOFL1 and AtSOFL2 in plant development, the double mutant *SOFL1-RNAi62 sofl2-1* was generated. Analysis of this double mutant uncovered a role for AtSOFL1 and AtSOFL2 in the regulation of cytokinin metabolism.

### Overexpression of *AtSOFL1* and *AtSOFL2* Leads to Elevated Endogenous Cytokinin Levels and Corresponding Phenotypes

The overexpression of *AtSOFL1* or *AtSOFL2* confers phenotypes including reduced apical dominance, smaller leaves, underdeveloped root system and delayed senescence (Figure 1). These phenotypes are similar to those of transgenic plants expressing the *Agrobacterium tumefaciens ipt* gene, encoding the enzyme for the first step of cytokinin biosynthesis [35], [36]. Endogenous cytokinin measurements confirmed that the *AtSOFL1-* and *AtSOFL2*-overexpressing plants are cytokinin-overproducing lines (Table 1). The *35S:AtSOFL1* and *35S:AtSOFL2* seedlings accumulated significantly higher levels of tZR and iP7G in both aerial parts and roots. Increased levels of iPRMP as well as tZ7G and cZ7G were also detected in the aerial tissue and roots, respectively (Table 1). tZR and iPRMP are important components of the cytokinin biosynthetic pathway and can be easily converted to free-base cytokinins. To determine what caused the increased levels of tZR and iPRMP, we analyzed transcript accumulation of cytokinin biosynthesis *AtIPT* genes (*AtIPT1*-*AtIPT9*) in *35S:AtSOFL1* and *35S:AtSOFL2* transgenic plants. Unlike the *sob5-D*, no obvious change was observed in these plants (data not shown), implying that the function of *AtSOFL1*and *AtSOFL2* is different from that of *SOB5* gene. The increased levels of tZ7G, cZ7G and iP7G suggest that cytokinin glycosylation has been stimulated in these transgenic plants, perhaps as a means of balancing active cytokinin levels. In addition, the *35S:AtSOFL1* and *35S:AtSOFL2* seedling phenotypes could be largely phenocopied by culturing wild-type seedlings in the presence of cytokinin (Figure 1E, F). Together, these results support the hypothesis that AtSOFL1 and AtSOFL2 are involved in positively regulating specific cytokinin levels in *Arabidopsis*.

The *35S:AtSOFL1* and *35S:AtSOFL2* transgenic plants also exhibit phenotypes observed for mutants overexpressing plant *ipt* genes. For example, seedlings with estradiol-induced expression of *AtIPT8* (also known as *PGA22*) have shorter roots [37] and the overexpression of *Sho* in petunia and tobacco (*Nicotiana tabacum*) results in reduced apical dominance and delayed senescence. The *35S:AtSOFL1* and *35S:AtSOFL2* expressing plants also display similar seedling and adult phenotypes (Figure 1). Some plant-*ipt*-overexpressing mutant phenotypes, such as greater number of branches and leaves, are not observed in *35S:AtSOFL1* and *35S:AtSOFL2* expressing plants. This discrepancy could result from the accumulation of different levels of specific cytokinins in these mutants and/or the use of different plant species.

### AtSOFL1 and AtSOFL2 Function Redundantly as Positive Regulators in Cytokinin Metabolism

To further investigate the role of *AtSOFL1* and *AtSOFL2 in planta*, the *SOFL1-RNAi62 sofl2-1* double mutant was analyzed for its endogenous cytokinin content. Opposite to the increased cytokinin content of the *AtSOFL1-* or *AtSOFL2-*overexpressing plants, the most significant change in the double mutant was the decrease of specific cytokinin levels (Table 2, Table S3). The content of cytokinin nucleotides, tZRMP and iPRMP, in the double mutant was only 5% and 3% of the content in the wild-type control, respectively (Table 2). The level of iPRMP was increased in the *AtSOFL1-* and *AtSOFL2-*overexpressing transgenic plants, but decreased in the *SOFL1-RNAi62 sofl2-1* double mutant (Table 1, Table 2, Table S3), suggesting that the products of these two genes act as positive regulators of the endogenous level of iPRMP. *AtSOFL1* and *AtSOFL2* might also be positive regulators for the level of tZRMP. Compared with the double mutant, however, the content of the tZR instead of tZRMP was increased in the *35S:AtSOFL1* and *35S:AtSOFL2* transgenic plants (Table 1). Taking into consideration that conversion between tZRMP and tZR is extremely fast [6], this discrepancy could be explained by the assumption that the overexpression of *AtSOFL1* and *AtSOFL2* in *Arabidopsis* probably also affected the conversion between tZRMP and tZR.

To sum up, our data clearly indicate that AtSOFL1 and AtSOFL2 play an important role in regulating endogenous cytokinin nucleotide levels of *Arabidopsis* plants. Kurakawa *et al*. [13] demonstrated that, in meristems, the fine-tuning of active cytokinin levels is fulfilled by directly converting inactive cytokinin nucleotides to their free-base forms. Their results suggest that the interconversion between the active forms and their corresponding nucleotides is one of the mechanisms plants use to maintain the optimally balanced levels of active cytokinins. It is possible that in the *SOFL1-RNAi62 sofl2-1* mutant some steps in the cytokinin biosynthetic pathway are suppressed and cytokinin nucleotides are preferentially converted to corresponding free bases to maintain the active cytokinin levels, which result in lower nucleotide levels. The slightly increased levels of cZ, cZ9G, iP7G and iP9G in the double mutant might be side effects of this balancing mechanism.

Although considerable success has been achieved in the past few years in understanding cytokinins' function and metabolic system [5], [6], [12], some mechanisms remain unknown. For example, the biological activity of cytokinin nucleotides is still unclear, since they usually show no activity in bioassays but can bind to certain cytokinin receptors [38]. High concentrations of extracellular cytokinin nucleotides have been detected in culture media of the bryophyte *Physcomitrella patens* as well as in tobacco cell suspension culture media [39], [40]. However, the mechanism behind this phenomenon and its significance are unknown. Further investigations into the role of cytokinin nucleotides and their metabolic pathways may provide a deep insight into the changes of cytokinin content in the *SOFL1-RNAi62 sofl2-1* mutant.

### The Possible Role of AtSOFLs in Cytokinin-Mediated Development

In the *SOFL1-RNAi62 sofl2-1* mutant, only the levels of specific cytokinins were affected. The content of active cytokinins (the content of iP is below our detection limit) was not significantly changed (Table 2), implying that cytokinin homeostasis was not disturbed in this mutant and that AtSOFL1 and AtSOFL2 are probably involved in the fine-tuning of cytokinin content. This could also explain the lack of obvious adult mutant phenotypes for the *SOFL1-RNAi62 sofl2-1* plants.

To further investigate the role of AtSOFL1 and AtSOFL2 in plant development, the sensitivity of the *SOFL1-RNAi62 sofl2-1* seedlings to exogenous cytokinins was examined with callus-formation and inhibition-of-hypocotyl-elongation assays and the results suggest that the sensitivity of the double mutant to exogenous cytokinin is decreased (Figure 5, Figure 6). One possible explanation is that AtSOFL1 and AtSOFL2 also act as positive regulators in cytokinin signaling. Interestingly, *sob5-D* also showed decreased cytokinin sensitivity [27], which may result from the inhibitory side effect of the supra-optimal cytokinin concentrations in the mutant.

The spatial and temporal distribution of bioactive cytokinin levels is strictly controlled in plant development. Although some enzymes have been found to be involved in the fine-tuning of cytokinin concentrations [13], the exact mechanism is not completely clear. Here we have shown that AtSOFL1 and AtSOFL2 play a role in the fine control of cytokinin levels. Both overexpression and loss-of-function analysis demonstrate that AtSOFL1 and AtSOFL2 are positive regulators of the content of specific cytokinins. Since different cytokinin species have been proposed to have distinct physiological activities [2], [41], the regulation of specific cytokinin levels could be an effective way of providing specific types of cytokinins when they are needed to achieve the optimal growth in different developmental processes.

## Materials and Methods

### Plant Material and Growth Conditions

All plants used in this study are *Arabidopsis thaliana* ecotype Columbia (Col-0). Seeds were surface-sterilized and cold-treated as described in Ward *et al*. [42] and sown on 0.5× Murashige and Skoog (MS) medium (PhytoTechnology Laboratories Inc., Shawnee Mission, KS) containing 1.5% (w/v) sucrose, 0.8% (w/v) phytablend (Caisson Laboratories Inc., Rexburg, ID) with appropriate antibiotics or 0.5× MS medium containing 1.5% (w/v) sucrose and 1% (w/v) phytagel (Sigma-Aldrich, St. Louis, MO). Unless otherwise noted, the growth conditions for all seedlings and plants were the same as described by Ward *et al*. [42].

### RT-PCR Analysis

RT-PCR analysis was performed as described in Ward *et al.* [42]. The *ubiquitin10* (*UBQ10*) gene was used as an internal template control, which was amplified using 5′-GGT ATT CCT CCG GAC CAG CAG C-3′ and 5′-CGA CTT GTC ATT AGA AAG AAA GAG ATA ACA GGA ACG G-3′ as primers for 22 cycles. The *AtSOFL1* gene was amplified for 28 or 32 cycles using the following primers: 5′-GGC TGA TGA TGG TTA TGA GAA CGA TGA TGG C-3′ and 5′-TTT GCT CAC CTT CCC TCT ACT CTG GAC ACG-3′; the *AtSOFL2* gene was amplified for 26 or 30 cycles using the following primers: 5′-GAG GTA GAT GAT GAT GGT GAT GGT GAT GAA G-3′ and 5′-CTG TCA CGA ACT CTG TTC TCT GTC TGA-3′. All reactions were repeated three times with consistent results.

### Overexpression of AtSOFL1 and AtSOFL2

To generate the *35S:AtSOFL1* and *35S:AtSOFL2* constructs, the coding region of *AtSOFL1* and *AtSOFL2* were amplified by PCR using following primers: for *AtSOFL1*, 5′ - CGA TCT GGT ACC TCT TTC TGT TGC GTG AAG CCA TG - 3′ and 5′ - CCG CAT CTA GAC CCA TAT CTT CTT CTC TCA TTC GC - 3′; for *AtSOFL2*, 5′ - CGA TCT GGT ACC GTG TTA GCC ATG GAG TCT CCA AG - 3′ and 5′ – CCG CAT CTA GAA ACC AGT ATG AGT CTT ATT TGG T - 3′. Each PCR product was digested with *Kpn*I and *Xba*I and ligated into pCHF3 cut with the same restriction enzymes. pCHF3 is a binary vector carrying the cauliflower mosaic virus 35S promoter and a pea ribulose 1,5-bisphosphate carboxylase/oxygenase terminator, and conferring kanamycin resistance for selection in plant [43].

These two constructs were separately transformed into *Arabidopsis* (Col-0) according to the floral dip method [44] using *Agrobacterium tumefaciens* strain GV3101. Multiple transformants were identified by screening on plates containing 30 mg l-1 kanamycin. Homozygous lines were identified in the T3 generation. For each construct, at least three representative lines with obviously increased expression level of *AtSOFL1* or *AtSOFL2* (based on RT-PCR analysis described above) were chosen for detailed analysis.

### Overexpression and Point-Mutation Analysis of the SOFL Domain in AtSOFL2

To generate *AtSOFLN, AtSOFLD, AtSOFLCD* and *AtSOFLC* constructs, the corresponding DNA fragments were PCR-amplified from the *35S*:*AtSOFL2* construct using the following oligonucleotides: AtSOFLN, 5′-CCG TTC GGA TCC ATG GAG TCT CCA AGA ATT C-3′ (LNZ) and 5′-CGA TCT GGT ACC ATT ACT ACT ATC ATC ATC ATC G-3′ (LNF); for AtSOFLD, LNZ and 5′-CTT AAG GGT ACC ATT AGG CCA TGA GGA TGC ATC AGA AGT C-3′ (LDF); for AtSOFLCD, 5′-CGA TCA GGA TCC ATG AAC AAT GAA AGC GAT GAC TCA ATG-3′ (LCDZ) and 5′- CGA TCT GGT ACC TTA TTT GGT TTT GCT C-3′ (LCDF); for AtSOFLC, 5′-CCG CAT GGA TCC ATG CCT AGC ACT CAT CAG CC-3′ (LCZ) and LCDF. The oligonucleotides LNZ, LCDZ and LCZ were used to introduce a *BamH*I site and LCDZ, LCZ were also used to add the first methionine codon; the oligonucleotides LNF, LCDF and LDF were used to introduce a *Kpn*I site and LNF, LDF were also used to add the stop codon. Each DNA fragment was cloned into the *BamH*I and *Kpn*I sites of pSN1301 [45].

Site-directed point mutations were performed using QuikChange Mutagenesis Kit according to the manufacturer's instructions (Stratagene, Cedar Creek, TX, USA). The mutagenic primers were designed using Stratagene's web-based QuikChange® Primer Design Program (http://www.stratagene.com/sdmdesigner/default.aspx): for T21I, 5′- AGT AGT TGC GAA TCA GGA TGG ATT ATG TAC ATA GAA GAC ACC TTT C-3′ and 5′- GAA AGG TGT CTT CTA TGT ACA TAA TCC ATC CTG ATT CGC AAC TAC T-3′; for D52N, 5′- TGA TGG TTT CTC TGT AAA AGA GGT AGA TAA TGA TGG TGA TGG TG-3′ and 5′- CAC CAT CAC CAT CAT TAT CTA CCT CTT TTA CAG AGA AAC CAT CA-3′; for D80N, 5′- GAT GAC TCA ATG ACT TCT AAT GCA TCC TCA TGG CCT A-3′ and 5′- TAG GCC ATG AGG ATG CAT TAG AAG TCA TTG AGT CAT C-3′. Each amino acid substitution was confirmed by sequencing.

These constructs were separately transformed into *Arabidopsis* (Col-0) according to the floral dip method [44] using *Agrobacterium tumefaciens* strain GV3101. Multiple transformants were identified by screening on plates containing 30 mg l^−1^ kanamycin and homozygous lines were obtained in T3 generation. For each construct, three representative lines were chosen for detailed analysis.

### Generation of the GUS Fusion Construct and Plant Transformation

A 1811-bp DNA fragment containing the *AtSOFL1* coding sequence and 890 bp genomic DNA upstream of the gene was amplified by PCR using the following primers: 5′-GTC CGC TGC AGA GTC ACA CAC ACA CAC ATA GGG CA-3′ and 5′-GTC GCT CAG ATC TTT GCT CAC CTT CCC TCT ACT CTG-3′. Similarly, a 1530-bp DNA fragment containing the *AtSOFL2* coding sequence and 1059 bp genomic DNA upstream of the gene was amplified by PCR using the following primers: 5′-GTC CAC TGC AGC CCA CAC TTG CTT TGC TTA GCT GT - 3′ and 5′ – GTG GCT CAG ATC TTT GCT CAC CTT GAC ACG ACT AGC - 3′. Each PCR product was digested with *Pst*I and *Bgl*II to create a translational fusion with the *GUS* gene in the pCAMBIA1301 binary vector (CAMBIA, Canberra, Australia), resulting in fusion construct *AtSOFL1:AtSOFL1-GUS* and *AtSOFL2:AtSOFL2-GUS*. The C-terminal fusion to GUS resulted in the loss of two N-terminal amino acids of GUS and two C-terminal amino acids of AtSOFL1 and AtSOFL2, respectively. Sequencing of these two constructs was performed to ensure that there were no errors in the open reading frame of the fused gene. *Arabidopsis* Col-0 plants were transformed with the *AtSOFL1:AtSOFL1-GUS* or *AtSOFL2:AtSOFL2-GUS* construct using the floral dip method. [44]. Homozygous, single-locus-insertion lines were identified in the T3 generation by screening on plates containing 20 mg l^-1^ hygromycin.

### GUS Staining

GUS staining was performed as described in Zhang *et al.* [27]. Plant tissues were incubated overnight in the following reaction solution: 100 mM sodium phosphate, pH 7.0, 10 mM EDTA, 0.5% (v/v) Triton X-100, 0.5 mM potassium ferrocyanide, 0.5 mM potassium ferricyanide, 1 mM 5-bromo-4-chloro-3-indolyl-β-D-glucuronide (X-GlucA, Research Products International Corp., Mount Prospect, IL). After staining, samples were treated with 70% (v/v) ethanol and 100% (v/v) ethanol to remove chlorophyll. Tissues were photographed in the same way as described in Turk *et al.* [46]. Multiple homozygous, single-locus-insertion transgenic lines with wild-type adult phenotypes for the *AtSOFL1:AtSOFL1-GUS* or *AtSOFL2:AtSOFL2-GUS* construct were used in this analysis. At least three transgenic lines containing the same construct showed similar GUS staining patterns. Plants and seedlings that were not transformed with the GUS reporter fusion showed no GUS activity.

### Isolation of the T-DNA Insertion Mutant *sofl2-1*

The T-DNA insertion mutant *sofl2-1* was obtained from the ABRC with stock number SALK_101699. To identify homozygous lines, the following primers were used: LBa1, 5′-TGG TTC ACG TAG TGG GCC ATC G-3′; LP, 5′-TCG TAA ACA CGT TGC TTT TTC AGT GGC-3′; and RP, 5′-TAA GAG GTT GGA ACA AGC TGA GGT ACG-3′. The primer pair of LBa1 and RP specifically amplifies DNA from the insertion alleles, and the primer pair of LP and RP was used for identification of the non-insertion alleles.

### Construction of the pSOFL1-RNAi Construct and Plant Transformation

For the pRNAi-SOFL1 construct, a 471-bp fragment corresponding to the C-terminal region of AtSOFL1 was amplified using primer pair 5′-CGA TGT GGT ACC GAA GAG AGT GAT GAT TCC ATG GCT TC-3′ (L1K1), 5′-GTC CAT CTC GAG CAA GGT ACA TAT TAT ATA ACA GGG T-3′ (L1X1) and 5′-GCA CGG ATC GAT GAA GAG AGT GAT GAT TCC ATG GCT T-3′ (L1C2), 5′-CCG CCT CTA GAC AAG GTA CAT ATT ATA TAA CAG GGT TC-3′ (L1X2). These PCR fragments were cloned into the pKANNIBAL vector (CSIRO Plant Industry, Victoria, Australia) in the sense direction with *Xho*I and *Kpn*I, and in the antisense direction with *Xba*I and *Cla*I. The resulting construct was cut with *Not*I and subcloned into the *Not*I site of binary vector pART27 (CSIRO Plant Industry, Victoria, Australia).

The pRNAi-SOFL1 construct was transformed via floral dip method into wild-type plants and homozygous *sofl2-1* lines that have completely lost their kanamycin resistance, respectively [44]. Transgenic plants were initially identified by screening on plates containing kanamycin (30 mg l^−1^). The presence of the transgenes was confirmed by PCR. Multiple homozygous, single-locus-insertion lines were identified in the T3 generation and at least three representative lines with obvious decreased expression level of *AtSOFL1* (based on RT-PCR analysis described above) were chosen for further analysis.

### Cytokinin Quantification

The cytokinin quantification was performed as described in Zhang *et al*. [27]. Each sample from three independent experiments was injected at least twice. The *SOFL1-RNAi62 sofl2-1* mutant and wild-type control (Col-0) were either grown on half-strength MS medium for 2 weeks or in soil for 3 weeks under short-day conditions (8 h light/16 h darkness) and 4 weeks under long-day conditions (16 h light/8 h darkness). Cytokinins were extracted from either 0.5 g of seedling tissues (aerial or root parts) or 1 g of young leaves according to Dobrev and Kamínek [47]. LC-MS analysis was performed in MS/MS mode by ion-trap mass spectrometer LCQ (Finnigan, USA) equipped with electrospray interface.

### Cytokinin Response Assays

Determination of the effect of cytokinin on callus formation was performed as described by Kubo and Kakimoto [32]. The upper part of hypocotyls were aseptically excised from 9-day-old seedlings and then cultured under continuous illumination with white light (100 µmol m^−2^ sec^−1^) for 24 days on GM medium [4.33 g l^−1^ Murashige and Skoog basal salts (PhytoTechnology Laboratories Inc., Shawnee Mission, KS, USA), 1% w/v sucrose, 10 ml l^−1^ 5% 2-(N-morpholino)ethanesulfonic acid (pH 5.7, adjusted with KOH), 1 ml l^−1^ Gamborg vitamin solution (PhytoTechnology Laboratories Inc.), 0.3% w/v phytagel (Sigma Aldrich, St Louis, MO, USA)] supplemented with 10 ng ml^−1^ 2,4-D and varying concentrations of kinetin. DMSO concentration in the plates was below 0.1% v/v. The experiment was repeated three times with consistent results.

The inhibition-of-hypocotyl-elongation assay was performed as described by Hutchison *et al.* [33]. Seeds were grown on horizontal plates containing 1×MS salts, 1% sucrose and 0.8% phytablend (Caisson Laboratories Inc., Rexburg, ID) with the appropriate concentration of benzyladenine (BA) for 6 days in the dark. Seedlings were photographed and measured using ImageJ 1.29J (NIH, Bethesda, MD, USA; http://rsb.info.nih.gov/ij/javal.3.1). Wild-type Col-0 plants were grown on each plate. The data represent the mean value of at least 25 individual seedlings expressed relative to the mean hypocotyl length of the same genotype grown without BA. The experiment was repeated three times with consistent results.

## Supporting Information

Figure S1 RT-PCR analysis of *AtSOFL2* mRNA accumulation in the *sofl2-1* mutant. (A) Primers used for RT-PCR. The gray box represents the coding region of the *AtSOFL2* gene. The black boxes indicate the position of the two conserved motifs among SOFL proteins. The large vertical arrow indicates the site of T-DNA insertion for the *sofl2-1* mutant and small horizontal arrows represent the site and orientation of the primers amplifying the *AtSOFL2* gene. (B) Total RNA was isolated from seedlings grown in continuous white light for 5 days. PCR was performed on cDNA by amplifying with *AtSOFL2*-specific primers for 30 cycles. The *ubiquitin10* (*UBQ10*) cDNA was amplified for 22 cycles and used as a control to normalize the amount of cDNA in the samples.
(0.04 MB DOC)

Figure S2 RT-PCR analysis of *SOFL1-RNAi* lines. Total RNA was isolated from *SOFL1-RNAi* seedlings (*SOFL1-RNAi-6*, R1; *SOFL1-RNAi-10*, R2; *SOFL1-RNAi-14*, R3) and the wild type control (Col-0) grown under continuous white light for 5 days. PCR was performed on cDNA by amplifying with *AtSOFL1*-specific primers for 32 cycles and *AtSOFL2*-specific primers for 30 cycles. The *UBQ10* cDNA, amplified for 22 cycles, was used as control to normalize the amount of cDNA in each of the samples.
(0.10 MB DOC)

Table S1 Lateral root number for seedlings grown on MS medium with or without 0.1 µM benzyladenine (BA) (as shown in Figure 1F). The numbers in parentheses indicate the standard deviation.
(0.03 MB DOC)

Table S2 The *SOFL1-RNAi62* transgenic plants show no obvious changes of endogenous cytokinin content. Cytokinin quantification was performed with rosette leaves from plants that were grown under short-day growth conditions for 4 weeks and moved to long-day growth conditions for 4 weeks. The abbreviations are either defined in the text or in the legend of Table 1 and Table 2. The results are based on three independent experiments and resulting data are expressed as means ± standard error.
(0.04 MB DOC)

Table S3 The *SOFL1-RNAi sofl2-1* mutant lines had decreased endogenous levels for tZRMP and iPRMP. Cytokinin quantification was performed with rosette leaves from *SOFL1-RNAi sofl2-1* mutant lines (*SOFL1-RNAi35 sofl2-1* and *SOFL1-RNAi62 sofl2-1*) and wild-type (Col-0) plants that were grown under short-day growth conditions for 3 weeks and moved to long-day growth conditions for 4 weeks. tZRMP, *trans*-zeatin riboside monophosphate; iPRMP, *N* ^6^-(Δ^2^-isopentenyl)adenosine monophosphate. The results are based on three independent experiments and resulting data are expressed as means ± standard error. (*) equals P<0.5 and (**) equals P<0.05 from a Student's unpaired two-tailed *t* test comparing the mutant and the wild type plants.
(0.03 MB DOC)
