# Supplementary material for: AtSOFL1 and AtSOFL2 Act Redundantly as Positive Modulators of the Endogenous Content of Specific Cytokinins in Arabidopsis
Source: PLoS One. 2009 Dec 9;4(12):e8236. doi: 10.1371/journal.pone.0008236 (PMC2785485; doi:10.1371/journal.pone.0008236)
Supplement: Figure S1 — RT-PCR analysis of AtSOFL2 mRNA accumulation in the sofl2-1 mutant. (A) Primers used for RT-PCR. The gray box represents the coding region of the AtSOFL2 gene. The black boxes indicate the position of the two conserved motifs among SOFL proteins. The large vertical arrow indicates the site of T-DNA insertion for the sofl2-1 mutant and small horizontal arrows represent the site and orientation of the primers amplifying the AtSOFL2 gene. (B) Total RNA was isolated from seedlings grown in continuous white light for 5 days. PCR was performed on cDNA by amplifying with AtSOFL2-specific primers for 30 cycles. The ubiquitin10 (UBQ10) cDNA was amplified for 22 cycles and used as a control to normalize the amount of cDNA in the samples. (0.04 MB DOC) [file pone.0008236.s001.doc]

(A)

(B)

*AtSOFL2*

*UBQ10*

Col-0*sofl2-1*

No

template

**Figure S1.** RT-PCR analysis of *AtSOFL2* mRNA accumulation in the *sofl2-1* mutant. (A) Primers used for RT-PCR. The gray box represents the coding region of the *AtSOFL2* gene. The black boxes indicate the position of the two conserved motifs among SOFL proteins. The large vertical arrow indicates the site of T-DNA insertion for the *sofl2-1* mutant and small horizontal arrows represent the site and orientation of the primers amplifying the *AtSOFL2* gene. (B) Total RNA was isolated from seedlings grown in continuous white light for 5 days. PCR was performed on cDNA by amplifying with *AtSOFL2*-specific primers for 30 cycles. The *ubiquitin10 (UBQ10)* cDNA was amplified for 22 cycles and used as a control to normalize the amount of cDNA in the samples.
