# Supplementary material for: AtSOFL1 and AtSOFL2 Act Redundantly as Positive Modulators of the Endogenous Content of Specific Cytokinins in Arabidopsis
Source: PLoS One. 2009 Dec 9;4(12):e8236. doi: 10.1371/journal.pone.0008236 (PMC2785485; doi:10.1371/journal.pone.0008236)
Supplement: Figure S2 — RT-PCR analysis of SOFL1-RNAi lines. Total RNA was isolated from SOFL1-RNAi seedlings (SOFL1-RNAi-6, R1; SOFL1-RNAi-10, R2; SOFL1-RNAi-14, R3) and the wild type control (Col-0) grown under continuous white light for 5 days. PCR was performed on cDNA by amplifying with AtSOFL1-specific primers for 32 cycles and AtSOFL2-specific primers for 30 cycles. The UBQ10 cDNA, amplified for 22 cycles, was used as control to normalize the amount of cDNA in each of the samples. (0.10 MB DOC) [file pone.0008236.s002.doc]

*AtSOFL1*

*AtSOFL2*

*UBQ10*

Col-0 R1 R2 R3 R3 R3


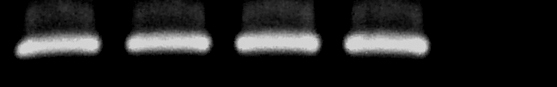

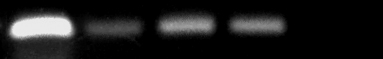


No

template

**Figure S2.** RT-PCR analysis of *SOFL1-RNAi* lines. Total RNA was isolated from *SOFL1-RNAi* seedlings (*SOFL1-RNAi-6*, R1; *SOFL1-RNAi-10*, R2; *SOFL1-RNAi-14*, R3) and the wild type control (Col-0) grown under continuous white light for 5 days. PCR was performed on cDNA by amplifying with *AtSOFL1*-specific primers for 32 cycles and *AtSOFL2*-specific primers for 30 cycles. The *UBQ10* cDNA, amplified for 22 cycles, was used as control to normalize the amount of cDNA in each of the samples.
