# Supplementary material for: AtSOFL1 and AtSOFL2 Act Redundantly as Positive Modulators of the Endogenous Content of Specific Cytokinins in Arabidopsis
Source: PLoS One. 2009 Dec 9;4(12):e8236. doi: 10.1371/journal.pone.0008236 (PMC2785485; doi:10.1371/journal.pone.0008236)
Supplement: Table S1 — Lateral root number for seedlings grown on MS medium with or without 0.1 µM benzyladenine (BA) (as shown in Figure 1F). The numbers in parentheses indicate the standard deviation. (0.03 MB DOC) [file pone.0008236.s003.doc]

**Table S1.** Lateral root number for seedlings grown on MS medium with or without 0.1 μM benzyladenine (BA) (as shown in Figure 1F). The numbers in parentheses indicate the standard deviation.

| Lateral root number (per seedling n>25) | | |
| --- | --- | --- |
|  | -BA | *+*BA |
| Col-0 | 11.6 (2.10) | 5.1 (1.32) |
| 35S:*AtSOFL1* | 4.7 (1.98) | — |
| 35S:*AtSOFL2* | 4.9 (1.45) | — |
