# Supplementary material for: AtSOFL1 and AtSOFL2 Act Redundantly as Positive Modulators of the Endogenous Content of Specific Cytokinins in Arabidopsis
Source: PLoS One. 2009 Dec 9;4(12):e8236. doi: 10.1371/journal.pone.0008236 (PMC2785485; doi:10.1371/journal.pone.0008236)
Supplement: Table S2 — The SOFL1-RNAi62 transgenic plants show no obvious changes of endogenous cytokinin content. Cytokinin quantification was performed with rosette leaves from plants that were grown under short-day growth conditions for 4 weeks and moved to long-day growth conditions for 4 weeks. The abbreviations are either defined in the text or in the legend of Table 1 and Table 2. The results are based on three independent experiments and resulting data are expressed as means ± standard error. (0.04 MB DOC) [file pone.0008236.s004.doc]

**Table S2.** The *SOFL1-RNAi62* transgenic plants show no obvious changes of endogenous cytokinin content.

| Cytokinin  Metabolites | Cytokinin content (pmol g^-1^ FW) | |
| --- | --- | --- |
|  | Col-0 | *SOFL1-RNAi62* |
| tZ | 1.40±0.76 | 1.70±0.51 |
| tZR | 1.50±0.26 | 2.43± 1.47 |
| tZRMP | 11.67±2.33 | 17.67±3.70 |
|  |  |  |
| cZ | 0.20±0.06 | 0.36±0.19 |
| cZR | 1.76±0.23 | 1.93±0.19 |
| cZRMP | 2.76±1.52 | 5.96±2.34 |
|  |  |  |
| iP | 1.30±0.31 | 0.80±0.36 |
| iPR | 3.63±0.47 | 5.50±0.85 |
| iPRMP | 7.73±0.76 | 9.80±0.60 |
|  |  |  |
| tZ7G | 59.00±2.00 | 53.67±6.33 |
| tZ9G | 19.33±4.33 | 17.66±5.45 |
| tZROG | 3.93±0.28 | 3.73±0.47 |
| cZ9G | 0.17±0.14 | 0.12±0.10 |
| DHZ7G | 9.23±0.90 | 9.06±0.87 |
| iP7G | 10.50±1.89 | 9.67±0.72 |
| iP9G | 0.36±0.20 | 0.46±0.03 |

Cytokinin quantification was performed with rosette leaves from plants that were grown under short-day growth conditions for 4 weeks and moved to long-day growth conditions for 4 weeks. The abbreviations are either defined in the text or in the legend of Table 1 and Table 2. The results are based on three independent experiments and resulting data are expressed as means ± standard error.
