# Supplementary material for: AtSOFL1 and AtSOFL2 Act Redundantly as Positive Modulators of the Endogenous Content of Specific Cytokinins in Arabidopsis
Source: PLoS One. 2009 Dec 9;4(12):e8236. doi: 10.1371/journal.pone.0008236 (PMC2785485; doi:10.1371/journal.pone.0008236)
Supplement: Table S3 — The SOFL1-RNAi sofl2-1 mutant lines had decreased endogenous levels for tZRMP and iPRMP. Cytokinin quantification was performed with rosette leaves from SOFL1-RNAi sofl2-1 mutant lines (SOFL1-RNAi35 sofl2-1 and SOFL1-RNAi62 sofl2-1) and wild-type (Col-0) plants that were grown under short-day growth conditions for 3 weeks and moved to long-day growth conditions for 4 weeks. tZRMP, trans-zeatin riboside monophosphate; iPRMP, N 6-(Δ2-isopentenyl)adenosine monophosphate. The results are based on three independent experiments and resulting data are expressed as means ± standard error. (*) equals P<0.5 and (**) equals P<0.05 from a Student's unpaired two-tailed t test comparing the mutant and the wild type plants. (0.03 MB DOC) [file pone.0008236.s005.doc]

**Table S3.** The *SOFL1-RNAi sofl2-1* mutant lines had decreased endogenous levels for tZRMP and iPRMP.

| Cytokinin  metabolites | Cytokinin content (pmol g^-1^ FW) | | |
| --- | --- | --- | --- |
|  | Col-0 | *SOFL1-RNAi35 sofl2-1* | *SOFL1-RNAi62 sofl2-1* |
| tZRMP | 13.10±4.44 | 4.15±2.34* | 0.20±0.20** |
| iPRMP | 1.55±0.35 | 0.28±0.04** | 0.08±0.04** |

Cytokinin quantification was performed with rosette leaves from *SOFL1-RNAi sofl2-1* mutant lines (*SOFL1-RNAi35 sofl2-1* and *SOFL1-RNAi62 sofl2-1*) and wild-type (Col-0) plants that were grown under short-day growth conditions for 3 weeks and moved to long-day growth conditions for 4 weeks. tZRMP, *trans*-zeatin riboside monophosphate; iPRMP, *N^6^*-(Δ^2^-isopentenyl)adenosine monophosphate. The results are based on three independent experiments and resulting data are expressed as means ± standard error. (*) equals P < 0.5 and (**) equals P < 0.05 from a Student’s unpaired two-tailed *t* test comparing the mutant and the wild type plants.
